# Fungal sporocarps house diverse and host-specific communities of fungicolous fungi

**DOI:** 10.1038/s41396-020-00862-1

**Published:** 2021-01-11

**Authors:** Sundy Maurice, Gontran Arnault, Jenni Nordén, Synnøve Smebye Botnen, Otto Miettinen, Håvard Kauserud

**Affiliations:** 1 Section for Genetics and Evolutionary Biology, University of Oslo, Blindernveien 31, 0316 Oslo, Norway; 2 Norwegian Institute for Nature Research, Gaustadalléen 21, 0349 Oslo, Norway; 3 Finnish Museum of Natural History, University of Helsinki, P.O. Box 7, FI-00014 Helsinki, Finland

**Keywords:** Microbial ecology, Molecular evolution

## Abstract

Sporocarps (fruit bodies) are the sexual reproductive stage in the life cycle of many fungi. They are highly nutritious and consequently vulnerable to grazing by birds and small mammals, and invertebrates, and can be infected by microbial and fungal parasites and pathogens. The complexity of communities thriving inside sporocarps is largely unknown. In this study, we revealed the diversity, taxonomic composition and host preference of fungicolous fungi (i.e., fungi that feed on other fungi) in sporocarps. We carried out DNA metabarcoding of the ITS2 region from 176 sporocarps of 11 wood-decay fungal host species, all collected within a forest in northeast Finland. We assessed the influence of sporocarp traits, such as lifespan, morphology and size, on the fungicolous fungal community. The level of colonisation by fungicolous fungi, measured as the proportion of non-host ITS2 reads, varied between 2.8–39.8% across the 11 host species and was largely dominated by Ascomycota. Host species was the major determinant of the community composition and diversity of fungicolous fungi, suggesting that host adaptation is important for many fungicolous fungi. Furthermore, the alpha diversity was consistently higher in short-lived and resupinate sporocarps compared to long-lived and pileate ones, perhaps due to a more hostile environment for fungal growth in the latter too. The fungicolous fungi represented numerous lineages in the fungal tree of life, among which a significant portion was poorly represented with reference sequences in databases.

## Introduction

The fungal life cycle is dominated by an inconspicuous mycelial stage, where the mycelia grow hidden belowground or within various substrates. Most Basidiomycota (=Dikarya), produce macroscopic fruit bodies (hereafter referred to as sporocarps) at some point(s) in their lives, where karyogamy, meiosis and formation of meiospores occur. For these fungi, the sporocarp is a key structure of the life cycle, as it bears meiotic spores that are fundamental for dispersal and population persistency. Though not always reflecting phylogenetic relationships [1–3], fungi have evolved an extensive morphological diversity of sporocarps [4] that are adapted to different strategies for maximising reproductive effort [5].

Sporocarps are complex structures varying widely in size, shape, colour, persistency and odour. Among wood-decay fungi, which are important nutrient recyclers in forest ecosystems, some species have evolved tough and resistant long-lived (perennial) sporocarps that can persist for several years, while others produce short-lived (ephemeral) sporocarps lasting for only a few days or weeks [6, 7]. Some sporocarps can extend relatively far out from the substrate with a stalk or being bracket-shaped (pileate), while others are relatively simple and “crust-like” (resupinate) adhering to the surface of the substrate [2].

The fruiting stage is a vulnerable phase of the fungal life cycle, as sporocarps are easily damaged by drought, heat, frost, or eaten and destroyed by other organisms. Being highly nutritious [8], sporocarps host a large diversity of bacteria [9–11], they can be consumed by small mammals [12] and birds [13], and also provide refuge and resources to a wide diversity of invertebrates such as slugs, snails and worms, as well as arthropods, including insects and mites [14]. Many of the fungivorous organisms play an important role as vectors for the dispersal of hyphal fragments or spores, either attached to their bodies or are excreted undigested, as they are enriched in recalcitrant compounds like chitin [15].

Whilst studies have revealed obligate associations between insects and fungi [16–19], including highly specific associations, far less is known about the diversity and richness of other species of fungi residing inside sporocarps. These fungi, which feed on other fungi, are termed fungicolous [20, 21] but little is known about their diversity, occurrence and interactions with host fungi.

So far, about 1500 fungicolous fungal taxa have been recorded [22]. Considering a global fungal diversity estimated between about two and four million species [23], the number of fungicolous fungi is probably a gross underestimate. Among fungicolous fungi adapted to colonise sporocarps, some can themselves produce macroscopic sporocarps through a rapid life cycle [24], but many do not, which is probably why they have largely been overlooked, despite being widespread in nature. One such overlooked group is yeasts, which can also be found as endophytes in plants and trees [25, 26], lichens [27] as well as in tripartite interactions with parasitic fungi and mushrooms [28]. Apart from several taxonomic studies [29–33] and studies addressing the parasitic activity of fungicolous fungi (see ref. [22]), their broad diversity is largely unexplored and their ecological roles uncharacterised. There are, therefore, many unaddressed questions, such as: how common are such fungal–fungal associations? How diverse is the fungicolous fungal community associated with the sporocarp of particular species, and does the community composition vary among and between sporocarps of the same and different species? Do fungal–fungal co-occurrences arise from random opportunistic use of the sporocarps, or do they represent adaptation to the specific niche(s) each host offers?

One reason why these fundamental questions remain unresolved is that fungicolous fungi have mainly been studied using culture-dependent approaches and morphology-based identification [34], which provide a limited view of the diversity. DNA metabarcoding, based on high-throughput sequencing of amplified marker(s), has become a powerful approach to scrutinise complex fungal communities and does not require prior knowledge of the community composition. Recently, a DNA-based study by Koskinen et al. [9] revealed that soft and short-lived agaricoid sporocarps (of the orders Agaricales, Boletales and Russulales) house a high diversity of fungicolous fungi, but without any distinct host preference. The relatively short lifespan of fleshy agarics (compared to other annual fungi) could partially explain this lack of host specificity.

Sporocarp characteristics, as well as environmental variables such as climate, are potential drivers of fungicolous fungal community composition. The structural composition of sporocarps vary from a simple organisation (monomitic hyphal system) in ephemeral sporocarps, to a more complex structure (di- or trimitic hyphal system) in the persistent ones. In addition to sporocarp morphology and persistency, extensive differentiation in their biochemical composition may also explain differences in fungicolous fungal community composition. Further, short-lived, fleshy and soft sporocarps typically have a higher water content than the resistant long-lived sporocarps, which may favour germination and growth of fungicolous fungi. Together, these sporocarp-related traits may lead to a variable degree of host-specialisation among fungicolous fungi. There are indications that some fungicolous fungi are host-specific [35–38] and, therefore, constrained to the geographic ranges of their hosts.

The overarching aim of this study was to reveal the diversity of fungicolous fungi residing in sporocarps of wood-inhabiting fungi. This was done by DNA metabarcoding analyses of sporocarp tissue from eleven wood-decay fungi, using the rDNA ITS2 region as a target marker. We expected that a vast majority of the ITS2 sequences will be derived from the host sporocarp tissue, but that a minor fraction of non-host ITS2 sequences, representing fungicolous fungi, will vary systematically according to the hosts, reflecting different levels of colonisation. We hypothesised that the short-lived fleshy sporocarps with higher water content are a more suitable habitat for fungicolous fungi, thus resulting in a higher proportion of non-host ITS2 sequences and higher diversity of fungicolous fungi (H1-1). Alternatively, the long-lived resistant sporocarps may accumulate a higher diversity of fungicolous fungi since they are available for colonisation for considerably longer, up to several years (H1-2). Second, we asked how the diversity of fungicolous fungi varies according to sporocarp morphology. We hypothesised that resupinate sporocarps house a higher diversity of fungicolous fungi due to a larger surface area to volume ratio, thus providing relatively more area for colonisation of fungicolous fungi (H2). Next, we ask how species-specific fungicolous fungi are. Due to differences in physical structure and chemical composition of the polypore sporocarps, we expected numerous specific co-occurrences between fungal hosts and fungicolous fungi (H3). Finally, the abundance of fungal hosts, surveyed at local and regional scales, allowed us to investigate whether there is a correlation between host density and the species richness of fungicolous fungi. Assuming that some degree of host preference exists, we expected that widespread and abundant hosts, acting as small islands in the forest landscape, provide habitat and resources for a larger pool of fungicolous fungi compared to rare hosts (H4). According to island biogeography theory, reduced distance separating hosts (i.e., distance effect) may increase the colonisation rate of fungicolous fungi.

## Material and methods

### Sample collection

The sampling was conducted in October 2014 in an old-growth forest dominated by Norway spruce (*Picea abies*) in northeast Finland, Kuhmo, Issakka (62°38′42.4″N 30°47′47.8″E). Compared to southern Finland, forestry has been less intensive in the northeast, including the Kuhmo region, where intensive forestry also lasted over a shorter period, which is today reflected in a relatively large area of old-growth forest with high connectivity between the remaining patches of old-growth boreal forest. Consequently, this area is relatively rich in dead wood and represents a biodiversity hotpot for many wood-dependent organisms, including wood-decay fungi where several species fruit at the same time, allowing intra- and inter-species comparative studies. To understand the natural dynamics of fungicolous fungi, choosing an area of this kind is preferable and allows a comparison across several species while maintaining other environmental variables constant. In addition, to limit the effect of confounding variables attributed to substrate, landscape and seasonality, we collected sporocarps only from logs of *Picea abies*, in one area (20 ha) within a short time frame (2 days).

Due to their importance in dead wood decomposition, their conspicuous size and well-characterised species traits and ecologies, polypores are a good study system to investigate the natural dynamics of fungicolous fungi in old-growth forests. We targeted common and rare boreal species having dead wood of *Picea abies* as main resource, that fruit in a shared time frame and with sufficient occurrences thus allowing a comparative population-level study. Eleven wood-decay fungal species were collected: the polypores *Amylocystis lapponica* (amylap), *Antrodia serialis* (antser), *Gloeophyllum sepiarium* (glosep), *Fomitopsis* (*Rhodofomes*) *rosea* (fomros), *Fomitopsis pinicola* (fompin), *Phellopilus nigrolimitatus* (phenig), *Phellinidium ferrugineofuscus* (phefer), *Phellinus* (*Fuscoporia*) *viticola* (phevit), *Postia cyanescens* in *Postia caesia* complex (poscae), *Trichaptum abietinum* (triabi) and the corticioid species *Phlebia centrifuga* (phecen). For each of the focal species, we included 16 sporocarps (individuals) collected on different spruce logs (Fig. S1), totalling 176 sporocarps.

### Sample processing and DNA extraction

We processed both annual and perennial species similarly, by removing the outer surface layer to avoid aerial contaminants and processing the subiculum layer. Between 10 and 15 small pieces of ~5 mm^3^, each were cut from the subiculum layer, placed in 2-mL tubes containing 800 µl of 2% CTAB and 1% beta-mercaptoethanol, and stored at −20 °C until DNA extraction. The samples were then grinded 4 × 45 s at 25 oscillations s^−1^ with two tungsten-carbide beads (2 mm) using the Retsch MM200 mixer mill and stored at −80 °C overnight. Subsequently, DNA was extracted using a modified CTAB/chloroform extraction protocol [39, 40]. The DNA extracts were cleaned with an E.Z.N.A Soil DNA kit (Omega Biotek) by adding the HTR reagent and then following the manufacturer´s guidelines, and eluted into 100-µL elution buffer. For each sample, we quantified the DNA concentration using the Qubit dsDNA BR Assay kit (Life Technologies) and standardised with 10-mM Tris in the range of 5–10 ng µL^−1^.

### Preparation of metabarcoding libraries and sequencing

In addition to the 176 DNA extracts, twelve technical replicates (at least one sample for each species), two mock samples (artificial fungal community composed of six other fungal species) and two PCR-negative controls were added in the library preparation, resulting in a total of 192 samples processed in 2 × 96 PCR plates. Amplicon libraries were constructed using a combination of 96 uniquely tagged primers designed to target the variable ITS2 region, with the reverse primer ITS4 (5′-*x*CTCCGCTTATTGATATG from [41]) and modified forward primer gITS7 (5′-*x*GTGA*R*TCATCGA*R*TCTTTG from [42]), barcodes *x* ranging from 6 to 9 base pair. One microlitre of DNA template was used for the 25-µl PCR reaction containing 14.6 µl of Milli-Q water, 2.5 µl of 10x Gold buffer, 0.2-µl dNTPs (25 nM), 1.5 µl each of reverse and forward primers (10 µM), 2.5-µl MgCl_2_ (50 mM), 1.0-µl BSA (20 mg/ml) and 0.2-µl AmpliTaq Gold polymerase (5 U/µl). DNA was amplified as follows: initial denaturation at 95 °C for 5 min, followed by 25 cycles of denaturation at 95 °C for 30 s, annealing at 55 °C for 30 s, and elongation at 72 °C for 1 min, and a final elongation step at 72 °C for 10 min. Amplifications were assessed by electrophoresis on a 1.5% agarose gel, normalised using the SequalPrep Normalisation Plate Kit (Invitrogen) and eluted into a total volume of 20-μl Elution Buffer. The 96 PCR products within each plate were pooled, concentrated and cleaned using Agencourt AMPure XP magnetic beads (Nerliens Meszansky AS). Quality control was performed using ds DNA 1000 Bioanalyzer (Agilent Technologies) and Qubit (Life Technologies). The two libraries were barcoded with Illumina adapters, indexed with 20% PhiX and sequenced in one Illumina MiSeq (Illumina, San Diego, CA, USA) lane with 2*300-bp paired-end reads at StarSEQ (StarSEQ GmbH, Mainz, DE).

### Bioinformatics analyses

The entire raw dataset comprised 25,953,804 sequences. Sequencing errors, including substitutions and indels were corrected using bayeshammer software [43]. The resulting 25,935,562 reads were merged using pear [44], with a minimum of sequence length of 200 bp, and filtered by quality and length using Fastx-toolkit [45] (quality = 30, percentage = 90) and vsearch [46] (fastq_maxee = 0.5, fastqminlen = 100, fastq_truncqual = 21). Paired-end reads were demultiplexed using sdm software [47]. Both forward and reverse primers were removed using cutadapt [48] followed by ITS extractor program [49]. The ITS2 sequences were dereplicated and sorted by abundance using vsearch. Operational taxonomic units (OTUs) were clustered at 97% similarity and chimeras were removed with vsearch (abskew = 2, mindiffs = 3, mindiv = 0.8, minh = 0.28). As we wanted to restrict our analyses to the most abundant fungi, singletons were excluded, and only OTUs supported by a total observation count of >10 sequences in the dataset were retained. Taxonomy of the ITS2 representative sequences was assigned using BLAST in qiime [50] against the NCBI and unite databases [51] (v. 7_99_s_01.12.2017). The raw OTU table comprised 16,196,863 reads assembled into 2188 OTUs. To correct for over-splitting of OTUs due to e.g., PCR and sequencing errors, we processed the raw OTU table using the lulu algorithm [52] and retained 1632 OTUs. We removed the 12 PCR replicates from the dataset after checking the repeatability by DCA (Fig. S2). OTUs without corresponding blast hit and non-fungal OTUs (mainly Rhizaria), were excluded from the dataset (1385 OTUs retained). To minimise the proportion of tag-switching inherent to library construction, Illumina sequencing platforms and bioinformatics steps, we ran the owi_renormalized script (https://github.com/metabarpark/) with a 10% threshold. We subsequently removed the OTUs corresponding to the mock community composed of six fungal species, the two negative PCR controls and manually curated for tag-switching, thus removing the mis-assigned reads corresponding to the host species in other samples. The final filtered OTU table was composed of 1794,144 reads accounting for 1367 OTUs. We then used the *rrarefy* function in the R package vegan ([53], v 2.4-5) to make abundances comparable between samples (10,000 reads/sample) and excluded two samples (fompin3 and fompin4).

### Compilation of metadata

The focal fungal hosts are characterised by measurable traits that vary across species. A series of these trait variables were compiled from the literature [6, 7, 54, 55]. Life-history traits included (i) specialisation to main host tree species, and traits-related to sporocarp such as (ii) sporocarp lifespan (short- or long-lived), (iii) morphology (pileate or resupinate), (iv) sporocarp hyphal system (mono, di- or trimitic), (v) size, which were categorised in three classes, with 1 = small (up to the size of a fingertip for pileate or a few fingerprints for resupinate), 2 = intermediate (up to the size of an apple-half for pileate or a palm for resupinate) and 3 = large (larger than the size of a fist for pileate or two palms for resupinate); (vi) thickness (minimum and maximum) and (vii) average hymenophore surface area (J. Nordén, unpublished data). Abundance data were inferred at the (viii) forest scale and were registered as the number of logs with the focal species (local abundance) [56], (ix) regional abundance of the species [55] and finally, (x) the proportion of fungicolous fungal reads in each sample was estimated from the current dataset and inferred as a response variable.

### Statistical analyses

To estimate whether the sampling effort (*n* = 16 sporocarps per fungal host) reached an acceptable level of efficiency, we constructed species-accumulation curves for the 11 fungal hosts using the function specaccum in vegan with the method rarefaction (Fig. S3). The effects of sporocarp-related traits were evaluated using OTU richness and Shannon H index, both calculated per sporocarp, as incorporated in vegan and the visualisations were made with ggplot2 package v 2.2.1 [57]. To investigate the effect of sporocarp lifespan and morphology on OTU richness (log-transformed) and Shannon diversity, linear mixed effect models were fitted using LME4 package in R, with host species as a random effect. Full models were specified with all the explanatory variables, and to identify the most parsimonious model, the models were subjected to a backward elimination procedure based on Akaike information criterion.

To account for the phylogenetic relationship between hosts, we extracted partial 28S rRNA sequences for the 11 fungal species from NCBI, performed a multiple sequence alignment in muscle [58] and calculated a pairwise distance matrix based on the Maximum Composite Likelihood model in MEGA [59]. Likewise, we computed a distance matrix from the fungicolous fungal community, aggregated both matrices and performed a Procrustes analysis with 9999 permutations in vegan to correlate the two matrices.

To visualise the variation in the community composition of OTUs across species, we used non-metric multidimensional scaling (NMDS) implemented in vegan, with parameter settings as recommended by Liu et al. [60]. To confirm convergence of the NMDS, the two best solutions were compared using Procrustes comparisons with 999 permutations. Since it was not possible to produce NMDS ordinations with acceptable stress values and convergence, we reduced the dimensions of the data by removing 11 samples with <5 OTUs. In addition, we performed a DCA and tested for consistency with NMDS; correspondence of the results and absence of artefacts, such as arch-effect (NMDS), tongue effect (DCA) and absence of extreme outliers, were interpreted as reliable gradients found. The effects of sporocarp morphology (pileate vs. resupinate), lifespan (short vs. long-lived) and size, local abundance of the fungal host species (occurrence) and the proportion of fungicolous fungi were visualised using vectors from the envfit function in vegan. The fit (*R*^2^) of each variable to the NMDS was assessed with a Monte-Carlo analysis of 999 permutations (Table 1). Using variance partitioning, canonical correspondence analysis with 999 permutations, we quantified the independent components of variation in the fungal community composition explained by hosts, sporocarp-related traits (lifespan, morphology and size), abundance of hosts (local and regional) and proportion of fungicolous fungi. To reveal detailed information on the multiple responses of the fungal community to each variable, we applied Bayesian generalised linear modelling implemented in R-INLA [61], in which host species was employed as a factor to predict the proportion of fungicolous fungi. To investigate the effects of host species abundance (applied as log of counts data) at local and regional scales on the richness of fungicolous fungi, we estimated an additional alpha index, Chao1 using phyloseq [62] that accounts for rare OTUs in low abundance in the dataset.

**Table 1:** Goodness-of-fit statistics (*R*^2^) for variables with the NMDS axis explaining variations in fungicolous fungi community profiles.

Variables	*R*^2^	*p*
Host	0.555	0.001
Sporocarp characteristics	0.389	0.001
Proportion of fungicolous fungi	0.305	0.001
Host abundance (local)	0.042	0.05
Sporocarp size	0.363	0.001
Morphology	0.107	0.001
Lifespan	0.023	0.05

The significance was based on Monte-Carlo permutations. Sporocarp characteristics refer to thickness (minimum and maximum) and the hymenophore surface area.

To identify significant co-occurrence patterns between host fungi and fungicolous fungi (i.e., species that are significantly correlated in the dataset), we performed an indicator species analysis using the R package Indicspecies [63]. First, we applied the multipatt function to determine the list of OTUs that are correlated to a particular host. Second, based on a permutation test, we filtered the indicator value index to retain only significant associations (*p* value < 0.05) with at least two fungal host occurrences. We then used Cytoscape [64] to visualise the fungicolous fungi OTUs interaction networks among the eleven host fungi.

## Results

### Proportion and taxonomic composition of fungicolous fungi

**Fig. 1:**
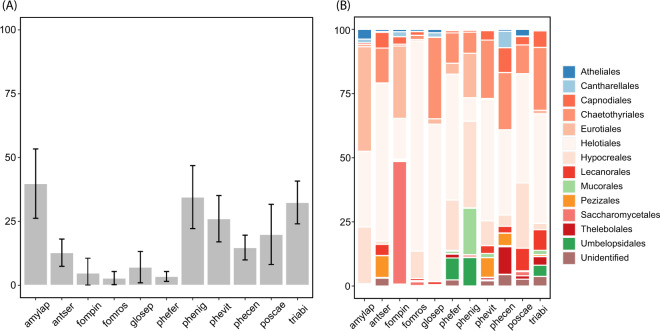
Proportion and taxonomic composition of the fungicolous fungi. **A** Bar plot representing the proportion of ITS2 sequences corresponding to non-host fungicolous fungi relative to all fungal sequences including the hosts. For each species, an average was calculated from 16 individual sporocarps and error bars represent 95% confidence intervals, indicating systematic differences across species. **B** Average proportion of 15 main orders (alphabetical listing) of fungicolous fungi, where blue colours represent Basidiomycota, red Ascomycota and green Mucoromycota.

Using a metabarcoding approach, we analysed the diversity of fungicolous fungi inhabiting sporocarps of eleven fungal host species collected within a forest. As expected, a large proportion of the ITS2 sequences corresponded to the fungal host species, while the relative abundances of non-host ITS2 sequences, representing fungicolous fungi, ranged from 2.8% in *Fomitopsis rosea* to 39.8% in *Amylocystis lapponica* (Fig. 1A). Most of the fungicolous fungi belonged to Ascomycota and varied between 77% in *Phellopilus nigrolimitatus* to 96% in *Antrodia serialis*, with Helotiales and Hypocreales as the most abundant orders (Fig. 1B). However, in the polypore *Fomitopsis pinicola*, ascomycete yeasts of Saccharomycetales were highly dominant, accounting for 47% of the fungicolous sequences. Basidiomycota, with 6.7% of the fungicolous reads, was the second most abundant phylum, with Atheliales and Cantharellales as predominant orders. Mucoromycota (6.3%) were present in variable proportions in eight of the focal hosts, mainly represented by the orders Mucorales and Umbelopsidales. In addition to these predominant phyla, we detected at lower frequencies (<1%) OTUs belonging to Chytridiomycota, Mortierellomycota, Olpidiomycota and Rozellomycota, mainly present in the hosts *Trichaptum abietinum* and *Phellinus viticola*.

### Fungicolous fungal diversity varies with life-history traits of host fungi

**Fig. 2:**
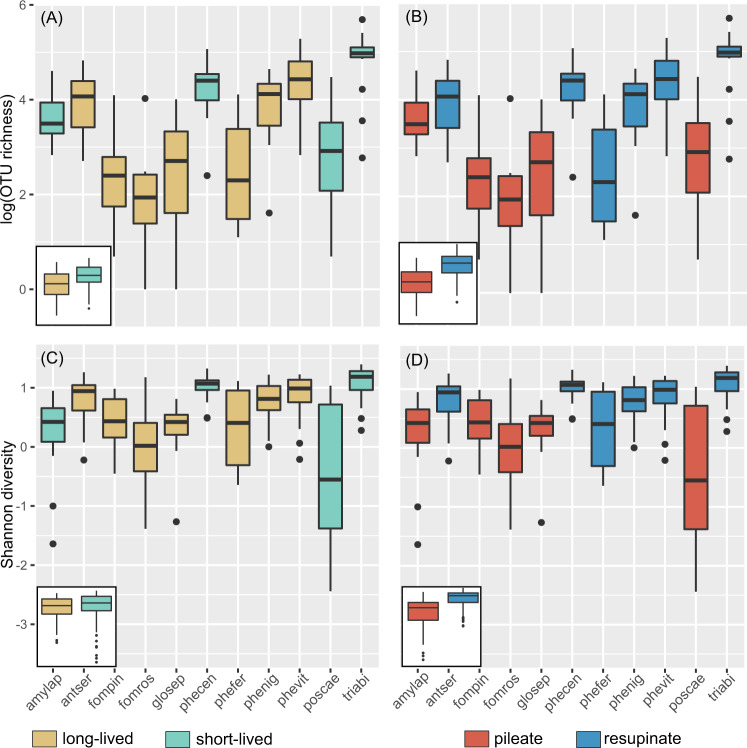
Alpha diversity of fungicolous fungal communities. Box plots illustrating variation in OTU richness (**A, B**) and Shannon diversity (**C, D**) of fungicolous fungi across 11 wood-decay fungal species. For each fungal host species, 16 sporocarps were analysed (excluding two fompin samples). The black dots represent data falling outside the interquartile range while the median is indicated by the dark line. The smaller frames (down left corner) compare long- and short-lived sporocarps (**A, C**) and pileate and resupinate sporocarps (**B, D**), respectively. Statistical differences between the different categories were evaluated using a Wilcoxon test with *p* value < 0.05.

The fungal host species were classified into two categories based on the lifespan of their sporocarps: short-lived (four species with 64 individuals) versus long-lived sporocarps (seven species with 110 individuals). A significant difference in OTU richness was observed between these two categories (Fig. 2A), with short-lived sporocarps hosting on average 2.58 (*p* = 0.03) more OTUs than the long-lived. In contrast, the Shannon diversity index was not significantly different between short- and long-lived sporocarps (*p* = 0.16, Fig. 2C). Among the short-lived species, *T. abietinum* (triabi) and *P. centrifuga* (phecen), producing resupinate sporocarps with large surface area, showed particularly high OTU richness. Regarding sporocarp morphology, we observed significantly higher OTU richness (estimate = 3.99 OTUs, *p* value = 0.004) as well as Shannon diversity index (estimate = 1.13 OTUs, *p* value = 0.001) in resupinate compared to pileate sporocarps. Species with pileate sporocarps showed higher variability in both OTU richness and Shannon diversity than resupinate species.

To test whether the diversity of fungicolous fungi is correlated to host abundance, we related the abundance data of the host species, both at the local and regional scales, against OTU richness, Shannon and Chao1 indices of fungicolous fungi (Fig. S4). We expected hosts with smaller population sizes to be associated with a lower diversity of fungicolous fungi. However, all correlations were weak and non-significant.

### Tentative drivers of the community pattern

**Fig. 3:**
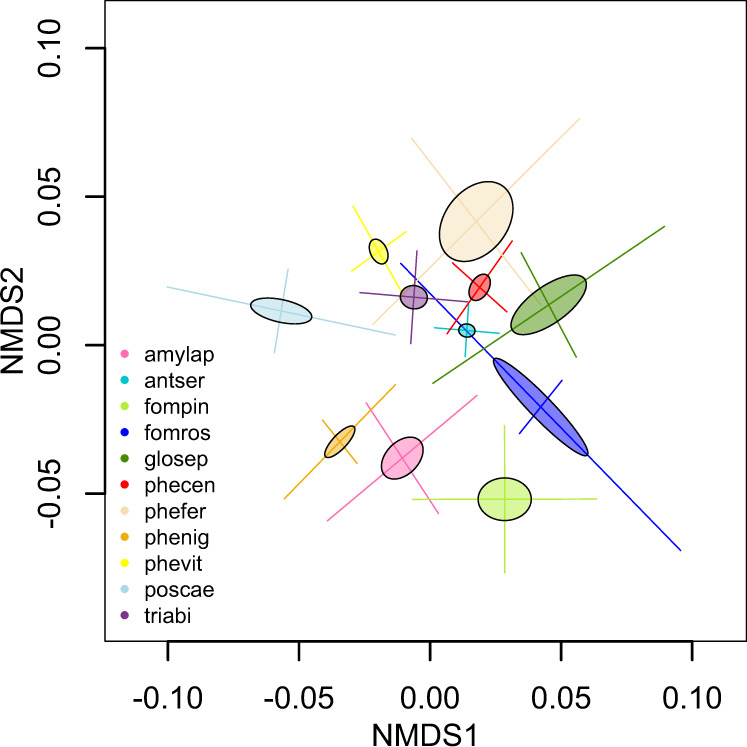
Non-metric multidimensional scaling (NMDS) plot based on Bray–Curtis dissimilarity matrix of fungicolous fungal community composition in 11 polypore species (in different colours) sampled within a forest. The ellipses reflect the standard error of the sample means and the bars represent the standard deviation. The explanatory variables with significant contribution to the constrained ordination are listed in Table 1. An NMDS plot displaying individual samples is included as Fig. S5.

Multivariate analyses indicated that all the assessed variables were significantly related to the compositional variation (Table 2). The analyses revealed that the fungicolous fungal communities were distinct between host species (Fig. 3, *R*^2^ = 0.56, *p* value = 0.001 and see Fig. S5 for individual sporocarps). Interestingly, species with long-lived sporocarps (e.g., *F. rosea* and *F. pinicola*) varied more in composition compared to many short-lived fungi (e.g., *A. serialis* or *P. caesia*), indicating that the ephemeral sporocarps host more species-specific fungicolous fungi than the perennial species (Fig. 3). Overall, the structure of the community composition clearly indicated strong associations between fungicolous fungi and fungal host species. Although non-significant (*p* = 0.19, *N* = 11), there was a high correlation (0.68) between the genetic distance of the host fungi, assessed using a 28S rRNA phylogeny, and the community distance of the fungicolous fungi.

**Table 2:** Richness analyses, with host as a random factor, intercept is log(average) for long-lived pileate fungi. Log(richness) with gaussian distribution.

	Value	Std. error	DF	*t* value	*p* value
(Intercept)	2.225	0.296	164	7.513	0.000
Lifespan: short-lived	0.948	0.362	8	2.617	0.030
Morphology: resupinate	1.383	0.350	8	3.949	0.004

### Fungal–fungal co-occurrences

**Fig. 4:**
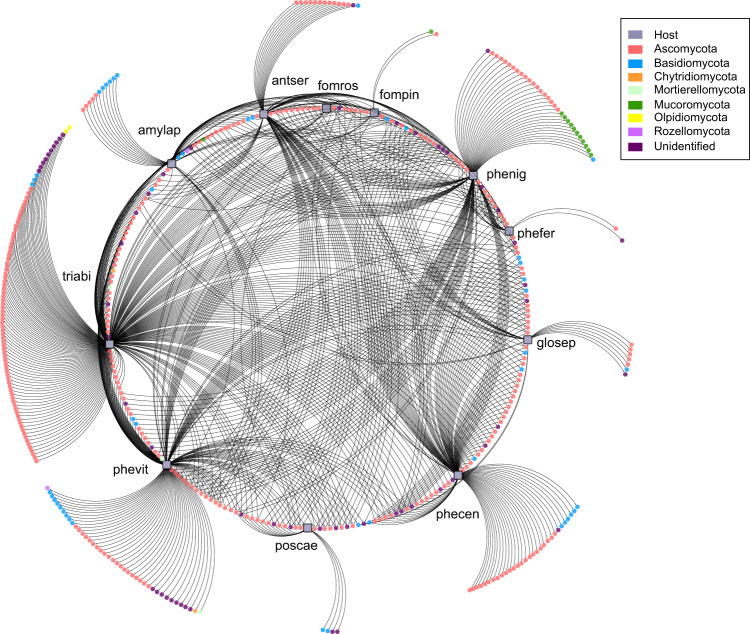
Network displaying 454 significant co-occurrences between 11 wood-decay fungal hosts and fungicolous fungi. Fungal hosts are represented by filled-square symbols while circles represent fungicolous fungal OTUs coloured according to phyla. The 236 OTUs of fungicolous fungi that are specific to a single host are located on the outer edge while 218 OTUs shared between two hosts or more are indicated on the inner edge. All edges represent a significant link (999 permutations test, *p* value < 0.05) between a host species and a fungicolous fungal OTU.

The correlation-based network shown in Fig. 4 illustrates how the fungicolous fungal OTUs were associated to the different fungal hosts. Out of the 454 fungal OTUs having significant associations (*p* < 0.01) with the eleven host fungi, 236 fungicolous OTUs were associated with a single host, whereas 218 OTUs co-occurred in at least two host species (up to 11 host species), the latter more likely reflecting generalists. The majority of the host fungi had species-specific associations with fungicolous fungal OTUs, with the exception of *Fomitopsis rosea*, which had only shared co-occurrences with other fungal hosts. In contrast, in four host species (*Phellopilus nigrolimitatus*, *Phlebia centrifuga*, *Phellinus viticola* and *Trichaptum abietinum*) we detected a high number (30–90) of species-specific fungicolous fungi that were not present in other hosts. At the same time, these species also shared the most fungicolous fungi with the other hosts, suggesting that these four hosts species harbour many specialists as well as generalists. Three hosts with short-lived sporocarps (*Amylocystis lapponica, Antrodia serialis* and *Gloeophyllum sepiarium)* were associated with a moderate number (7–14) of host-specific fungicolous OTUs. In general, species with smaller sporocarps and/or a smaller total hymenophore surface area per log have fewer specific associations, except for *P. ferrugineofuscus* and *F. pinicola*.

Among the species-specific OTUs, there was a large proportion of fungi without taxonomic assignment below the kingdom level, accounting for 13% of all the significant co-occurrences (52% unidentified OTUs at genus level). We could assign functions with some level of confidence to only a proportion of the identified OTUs. Of these, there were a few wood saprotrophs, including the brown rot *Calocera* sp. (Dacrymycetes) and the two white rotters *Dichostereum granulosum* and *Athelia decipiens*, all three species known to decay Norway spruce. In addition, several species known as soil saprotrophs in the orders Mortierellales and Mucorales were associated with long-lived sporocarps. The indicator species analysis supported numerous associations between *P. nigrolimitatus* and OTUs affiliated to Mucoromycota (*Umbelopsis* and *Mucor*). Besides fungal species known to be associated with plants (endophytes, mycorrhizal fungi and plant pathogens), we also detected fungal pathogens of animals, which were mainly from the class Leotiomycetes (Cryptomyces, Cryptosporiopsis, Leconeurospora and Pseudogymnoascus). Fungi previously reported to be associated with other fungi (facultative and obligate), formed both specific and generalist associations with the hosts. Endomycete yeasts described as parasites e.g., classes Microbotryomycetes and Tremellomycetes, made up a large proportion of the Basidiomycota, particularly in the hosts *P. caesia* and *G. sepiarium*. Several Ascomycota (e.g., *Coleophoma empetri*, *Hypomyces spp., Tolypocladium spp*., *Talaromyces infraolivaceus*) reported as parasites of fungi were also present in many of the hosts.

## Discussion

This study is one of the very first providing a DNA-based overview of fungal communities residing within sporocarps of various fungal hosts. We unveiled a species-specific community of fungicolous fungi inside sporocarps of wood-decay fungi in the boreal forest ecosystem. A significant proportion of the fungicolous fungi was poorly represented with associated reference sequences in databases, indicating that this diversity has hitherto been poorly explored. Our results support fungicolous fungi being widespread across different lineages within the fungal kingdom [65, 66], and dominated by Ascomycota, most with unknown ecological functions.

### Are short-lived sporocarps more suitable habitat for fungicolous fungi?

In concordance with our first hypothesis (H1-1), we obtained evidence that short-lived sporocarps hosted more fungicolous fungi, both in terms of OTUs richness and Shannon diversity. Most likely, the fleshy, spongy and ephemeral sporocarps, with a higher moisture content, represent a more favourable niche, facilitating germination and growth of fungicolous fungi, as compared to tough perennial sporocarps that might be more resistant to colonisation by fungicolous fungi. Our alternative hypothesis (H1-2), that long-lived sporocarps accumulate a higher diversity due to longer exposure time, was not supported. Colonisation by fungicolous fungi may represent a neglected but strong selection pressure on fruit body evolution, giving rise to different fruiting strategies. Fungi with short-lived sporocarps, predominant in the fungal kingdom, have developed a ‘boom and bust’ strategy, involving rapid sporulation and little investment in structural and physical defence against fungicolous fungi, leading to rapid senescence [67]. The opposite strategy is the production of complex and resistant long-lived sporocarps, limiting the colonisation of fungicolous fungi. However, there is considerable variation in sporocarp longevity between those enduring for several months (e.g., *P. ferrugineofuscu*s) to several years (*P. nigrolimitatus*), which may partly explain the variation in OTU richness and Shannon diversity of fungicolous fungi observed among these species. Regardless of the fruiting strategies, the mycelia inside the wood are generally long-lived [68].

Compared to the other host species producing short-lived sporocarps, *Amylocystis lapponica* had a lower alpha diversity of fungicolous fungi. Yearly variation in climatic conditions and sampling time (e.g., late autumn) can directly influence the diversity and composition of fungicolous fungi in short-lived species, a plausible reason for the lower diversity detected in *A. laponi*ca. Interestingly, we detected a high abundance of *Hypomyces* sequences in *A. lapponica*, which could also be seen in the field as orange to reddish spots on the host sporocarps. *Hypomyces* is a diverse worldwide genus of Pezizomycotina, including mycoparasitic species altering the original colour and shapes of their hosts, e.g., *Amanita*, *Agaricus*, *Lactarius*, *Russula and Polyporales* [69–72]. In several patho-systems when species-specific parasites dominate, like *Hypomyces* in *A. lapponica*, this could lead to competitive exclusion of other parasites (see ref. [73]), resulting in low alpha diversity as observed in sporocarps of *A. lapponica*. In addition, the relatively high density of *A. lapponica* in Issakka forest could directly amplify the abundance of specific-specific parasites.

### Is sporocarp morphology of importance?

We found solid support for our second hypothesis (H2), implying that resupinate sporocarps house a higher diversity of fungicolous fungi compared to pileate species. Resupinate sporocarps possess a significantly higher surface area to volume ratio. Consequently, a larger surface area of the sporocarps is in contact with the wooden substrate in resupinate species, as well as exposed to air and wind, precipitation and (in-)vertebrates, which are potential vectors of fungicolous fungi [74]. Though we processed the subiculum layer of the sporocarps, nonetheless we cannot exclude that a proportion of these non-host fungi might only be present as spores or mycelium fragments, without real ecological roles within the host fungi.

### How species-specific are the fungicolous fungi?

As hypothesised, we observed a relatively high number of species-specific co-occurrences between the focal host fungi and fungicolous fungi (H3). The multivariate analyses also supported that the assembly process of fungicolous fungi in sporocarps is not a random process, but instead governed by species-specific filtering processes. Indeed, at both forest scale and broader landscape level, biogeographic processes affecting the host fungi may also impact the fungicolous fungi. Among the possible probabilistic filtering processes maintaining species-specific associations over broader scales are local habitat suitability in terms of abiotic factors within the limits of variation for growth of fungicolous fungi (e.g., climatic variables, moisture content inside hosts, pH), sporocarp abundance, nutrient availability in the sporocarps, host susceptibility (defence mechanisms) and the capacity of colonisation by fungicolous fungi (types of interaction).

To establish and grow, fungicolous fungi must be able to withstand the biochemical defences of the host fungi. Polypore fungi produce secondary metabolites, including triterpenoids, organic acids and volatile organic compounds, known for their wide range of biological activities such as antimicrobial, anti-inflammatory, cytotoxic, antioxidant, immunostimulant and anti-thrombin properties [75]. Adaption to the various secondary metabolites may lead to host specificity.

Striking specific co-occurrences were observed between the host *P. nigrolimitatus* and several OTUs of Umbelopsidales (*Umbelopsis ramanniana*, *U. isabellina* and *U. angularis*). Commonly isolated from rhizosphere soils worldwide [76], there is increasing evidence that species of *Umbelopsis* occur as root endophytes of boreal trees [77]. Either sporocarps of *P. nigrolimitatus*, commonly produced beneath large and well-decayed logs, enable the recruitment of soil fungi as a result of ground contact or *Umbelopsis* species may have biotrophic relationships with the long-lived sporocarps of *P. nigrolimitatus*.

### To what extent does host abundance matter?

Island biogeography theory suggests that remote islands host less biodiversity than connected islands purely due to dispersal limitations. Likewise, if there are species-specific associations between fungicolous fungi and polypore hosts, as we have seen, one might expect that host species with large populations and numerous sporocarps will harbour a higher richness of fungicolous fungi compared to rarer species. The availability of abundance data of the host species, measured as number of colonised substrates (logs) at local and regional scales, allowed us to test this hypothesis (H4). However, we observed no significant correlations between the abundance of hosts and OTU richness (all diversity indices) at either spatial scale. Hence, other factors than host abundance are more important in driving the diversity of fungicolous fungi. In addition to the focal host species being historically restricted to old-growth boreal forests and regionally more abundant in northeast Finland, the number of host species examined here (*n* = 11) might be too low to draw any meaningful conclusions on this result.

### Plausible ecological roles of fungicolous fungi?

We revealed a high diversity of fungicolous fungi, widely spread across different lineages in the fungal kingdom. Among the fungicolous fungi described, most are classified as mycoparasites, probably based on the symptoms (modification of colour and shape and reduced growth) observed on mushrooms in nature or in the farming industry [78, 79]. Though, it is tempting to speculate that they have different ecological roles, as mycoparasites, saprotrophs of dead fungal host tissue or even mutualists, but from our data it is not possible to clearly determine their life strategies. It is well known that many fungi living as plant endophytes have beneficial functions to their hosts, such as *Epichloë* species [80–83]. These fungi employ different mechanisms which protect their host plants from grazing [84], either by acting directly (release of growth promoter and secondary metabolites) or indirectly (modification of host physiology and nutrient balance). An interesting parallel would be if fungicolous fungi could also produce metabolites protecting the host sporocarps against grazing e.g., by invertebrates. One might expect such beneficial fungi to coevolve with the host fungus and develop host specificity. Given the relatively short lifespan of sporocarps of some fungi, one may ask why fungicolous fungi adapt to hosts with such fruiting strategy? Obviously, the ease of colonisation and growth, together with low level of host defence is plausible arguments. However, the reasons may be more complex as some fungicolous fungi may not be solely restricted to the colonisation of sporocarps. For instance, some invasive necrotrophs are also capable to penetrate host cell walls and grow within the vegetative mycelial [85], just as endosymbiotic bacteria dwell inside fungal mycelium [86]. In such cases, the whole life cycle of the host is parasitised.

## Further work and implications

Our findings raise questions about how widespread fungal–fungal co-occurrences are across different ecosystems. This study was conducted on eleven fungal host species within one forest. A further step would be to assess whether these co-occurrence patterns, between fungicolous fungi and their fungal hosts, are stable at broader geographic scales, and to what degree they are also influenced by site characteristics, e.g., climate and forest connectivity. If the observed co-occurrence patterns are stable across broader geographic scales, this will support a strong coevolution. Since the composition of secondary metabolites in the host sporocarps likely play an important role, another step would be to characterise the active secondary metabolites of the different host species. In this regard one might expect that long-lived sporocarps have a more complex profile of secondary metabolites, leading to higher resistance to fungicolous fungi compared to short-lived ones. Owing to their ecological importance in nutrient recycling, widespread distribution, large diversity of sporocarp characteristics and the production of broad secondary metabolites, polypore fungi are clearly a well-suited study system for investigating host specificity and fungal–fungal coevolution.

Another aspect that may play an important role in structuring the community of fungicolous fungi is their tentative interactions with arthropods. Colonisation by arthropods may shape the fungal community, either directly by vectoring fungicolous fungi or indirectly by altering the physical structure and chemical composition of the sporocarps. Arthropods very likely act as vectors, not only of the host fungi, but also of its associated fungicolous fungi. Considering the community composition of arthropods colonising the host sporocarps together with the fungicolous fungi may provide valuable information about the community assembly processes in the sporocarps.

## Supplementary information

Supplementary Material_SMaurice

## Data Availability

The MiSeq raw sequence data is available on the NCBI short read archive (SUB8582638) under Bioproject PRJNA680258. The scripts for performing the statistical analyses and generating the main and Supplementary fig. together with the OTU table, the metadata, intermediate files are deposited on Dryad 10.5061/dryad.573n5tb66. Supplementary Material is available for download on the ISME website.
