# Supplementary material for: Fungal sporocarps house diverse and host-specific communities of fungicolous fungi
Source: ISME J. 2021 Jan 11;15(5):1445–57. doi: 10.1038/s41396-020-00862-1 (PMC8115690; doi:10.1038/s41396-020-00862-1)
Supplement: Supplementary file 1 — Supplementary Material_SMaurice [file 41396_2020_862_MOESM1_ESM.docx]

**Supplementary Material**

**Fungal sporocarps house diverse and host-specific communities of fungicolous fungi**

Sundy Maurice^1^, Gontran Arnault^1^, Jenni Nordén^2^, Synnøve Smebye Botnen^1^, Otto Miettinen^3^, Håvard Kauserud^1^

^1^Section for Genetics and Evolutionary Biology, University of Oslo. Blindernveien 31, 0316 Oslo, Norway, ^2^Norwegian Institute for Nature Research, Gaustadalléen 21, 0349 Oslo, Norway, ^3^Finnish Museum of Natural History, P.O. Box 7, FI-00014 University of Helsinki, Finland.

**Corresponding author**

Dr. Sundy Maurice

Section for Genetics and Evolutionary Biology, University of Oslo. Blindernveien 31, 0316 Oslo, Norway

Email: [sundymaurice@gmail.com](mailto:sundymaurice@gmail.com)

**Supplementary Figures**

**
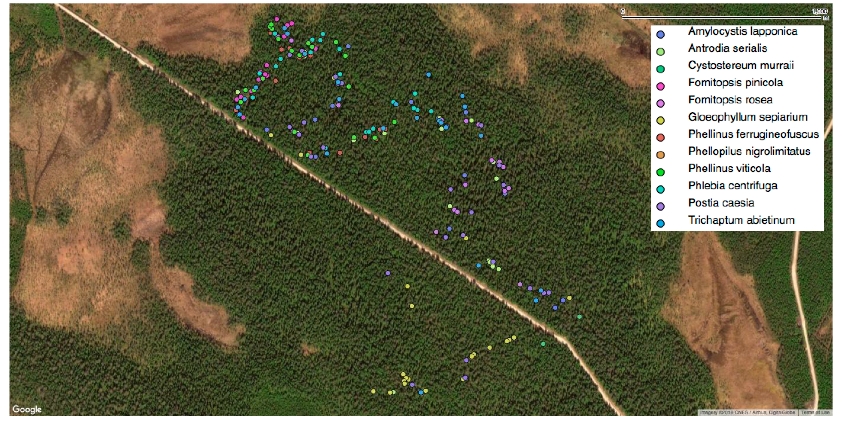
**

**Figure S1**. Sample collection map of the eleven wood-inhabiting fungal hosts in the Issakka forest. A unique 6-letter code was used for species. poscae: *Postia caesia;* phecen: *Phlebia centrifuga*; triabi: *Trichaptum abietinum*; phevit: *Phellinus viticola*; phefer: *Phellinidium ferrugineofuscus*; phenig: *Phellopilus nigrolimitatus*; amylap: *Amylocystis lapponica*; glosep: *Gloeophyllum sepiarium*; antser: *Antrodia serialis*; fompin: *Fomitopsis pinicola*; fomros: *Fomitopsis rosea.*

**

**Figure S2**. Multivariate analysis of fungicolous fungi in 11 replicates of fungal sporocarps as determined by metabarcoding of nrDNA ITS2. DCA (Detrended correspondence analysis) generated only from OTUs corresponding to fungicolous fungi (excluding the hosts OTUs). The pair of replicates is indicated by same colour, with one replicate for each of the 11 focal wood decay fungal species.

**Figure S3.** Accumulation curves of fungicolous fungi OTU richness captured from 16 sporocarps for the 11 wood-decay fungal. Curves and standard deviations were calculated from the rarified dataset (10 000 sequences/sample) using the *specaccum* function in vegan, with the method *rarefaction*.

**Figure S4**. Correlation between abundance (log of counts) of fungal sporocarps, surveyed at local (i.e forest-level as upper row) and regional (lower row) scales and alpha diversity (OTU richness, Shannon, and Chao1 diversity indices) of fungicolous fungi.

**Figure S5.** Nonmetric multidimensional scaling plot of all sequenced sporocarps, excluding the host OTUs. The NMDS distance matrix was calculated with Bray-Curtis index where each point represents a sample coloured by wood-decay host species.
